# Current Role and Future Prospects of Positron Emission Tomography (PET)/Computed Tomography (CT) in the Management of Breast Cancer

**DOI:** 10.3390/medicina60020321

**Published:** 2024-02-14

**Authors:** Nicole Robson, Dinesh Kumar Thekkinkattil

**Affiliations:** 1 Lincoln Medical School, Ross Lucas Medical Sciences Building, University of Lincoln, Lincoln LN6 7FS, UK; mzynr7@nottingham.ac.uk; 2 Lincoln County Hospital, Lincoln LN2 5QY, UK

**Keywords:** breast cancer, staging, PET CT, FDG PET, radiomics, artificial intelligence

## Abstract

Breast cancer has become the most diagnosed cancer in women globally, with 2.3 million new diagnoses each year. Accurate early staging is essential for improving survival rates with metastatic spread from loco regional to distant metastasis, decreasing mortality rates by 50%. Current guidelines do not advice the routine use of positron emission tomography (PET)-computed tomography (CT) in the staging of early breast cancer in the absence of symptoms. However, there is a growing body of evidence to suggest that the use of PET-CT in this early stage can benefit the patient by improving staging and as a result treatment and outcomes, as well as psychological burden, without increasing costs to the health service. Ongoing research in PET radiomics and artificial intelligence is showing promising future prospects in its use in diagnosis, staging, prognostication, and assessment of responses to the treatment of breast cancer. Furthermore, ongoing research to address current limitations of PET-CT by improving techniques and tracers is encouraging. In this narrative review, we aim to evaluate the current evidence of the usefulness of PET-CT in the management of breast cancer in different settings along with its future prospects, including the use of artificial intelligence (AI), radiomics, and novel tracers.

## 1. Introduction

Breast cancer is the most diagnosed cancer in women worldwide [1]. Furthermore, there are 685,000 related deaths yearly, making it the fifth leading cause of mortality worldwide [1]. The heterogeneous nature of the disease, with differing subtypes, increases the complexity of the disease; however, the combination of early detection programmes with improvements in the accuracy of staging and imaging techniques has increased the survival rates for breast cancer patients by enabling improved planning and treatment options compared to when surgery was the primary method of treatment [2]. At present, the five-year survival rate for women diagnosed within the UK is 85% when diagnosed at an early stage; however, this decreases to 26.6% when diagnosed at stage IV [3]. Thus, it is important to stage the patients accurately to ensure the best possible patient outcomes. The literature suggests that approximately 2–10% of breast cancers will be metastatic in nature at the time of diagnosis, with clear signs and symptoms permitting accurate diagnosis and treatment [4]. It is suggested that only 5–7% newly diagnosed breast cancer have occult metastasis [5,6,7]. In early breast cancer (T1 to T2), the incidence of distant metastases is <2% in comparison to more advanced tumours (T3 and T4) where it is as high as 15–20% [8,9,10]. Hence, the general consensus in most of the national and international guidelines, such as the National Institute for Care Excellence, the National Comprehensive Cancer Network (NCCN 2023), and the European Society for Medical Oncology (ESMO2023), is not to use routine staging to diagnose occult distant metastasis in early breast cancer patients without any specific symptoms [10,11,12]. There is a lack of a generalised consensus on indications as well as the type of staging investigations used in breast cancer management. Most centres use computed tomography (CT) of the thorax, abdomen, and pelvis, with a combination of other modalities such as magnetic resonance imaging (MRI) or bone scans. Currently, there are limited data on the usefulness of functional imaging modalities such as PET-CT scans as staging investigations in early and locally advanced breast cancer [13]. Current NCCN guidelines suggest that PET-CT may be helpful when standard imaging is equivocal and suggest that it may also be helpful in identifying unsuspected regional nodal disease or distant metastases when used in addition to standard tests. However, the guideline is not advisings its use in stages I and II and operable stage III breast cancer, as there is a high false-negative rate for the detection of subcentimetre lesions and low-grade disease and false positives in patients without locally advanced disease [11]. Its use in patients with stage III disease or when performing standard staging yields suspicious results, suggesting its main benefits lie in identifying unsuspected regional nodal disease and distant metastasis in locally advanced disease alongside standard staging [11]. However, there is increasing evidence for the usefulness of PET-CT in early-stage breast cancer [14,15]. Survival rates are 76–99% for locoregional metastases versus 20–28% for distant metastases, showing a decrease of 50% and therefore proving the importance of the accurate staging and early detection of cancer in increasing treatment options [1].

In addition to accurate staging, it is also important to assess tumour response to systemic treatment, which permits appropriate surgical treatment planning for breast cancer patients. Various studies have evaluated the role of PET-CT in assessing tumour response in order to tailor treatment options. Currently, the characterisation of tumour biology is dependent on invasive procedures such as tissue biopsy. However, sampling a lesion may not truly represent whole-tumour heterogeneity and it is almost impractical to carry out biopsies of every lesion, especially in the metastatic setting, to aid treatment planning. Radiomics is a rapidly evolving field of medical image analysis involving the extraction of quantitative metrics hidden within the pixels of medical images and routinely not visible to the human eye and studies have evaluated radiomic data from PET-CT in various settings. Likewise, artificial intelligence, which includes machine learning and deep learning, is also rapidly changing the scope of medical imaging.

In this narrative review, we aim to evaluate the current evidence of the usefulness of PET-CT in the management of breast cancer in different settings, along with its future prospects. 

## 2. Materials and Methods

The authors searched the MEDLINE and Pubmed databases for published peer-reviewed literature using the relevant MeSH terms of “PET-CT” OR “FDG-PET” AND “breast cancer” OR “breast” AND “radiomics” OR “artificial intelligence”. Articles published in English language were reviewed. Various levels of evidence were reviewed, such as randomised control trials, cohort studies, and case control studies. 

## 3. Results and Discussions

### 3.1. PET-CT in Primary Cancer Diagnosis

PET-CT is not a routinely used form of imaging in the detection of primary breast cancer. This is due to the high rate of false-negative results, especially with lesions less than 1 cm in size and with low-grade tumours [16]. Another major limiting factor is the higher cost involved with PET-CT. The sensitivity and specificity of PET-CT for the diagnosis of breast cancer is variable in different study settings and ranges from 48–96 and 73–100%, respectively [17]. Grueneisen et al., compared MRI, PET-CT, and PET-MRI in breast cancer patients. PET-MRI and MRI showed higher accuracy in identifying the tumour size than PET-CT (82%, 82% and 68%, respectively). This study also showed that both PET-MRI and MRI showed higher accuracy in detecting multifocal and multicentric breast cancer than PET-CT (89%, 89%, and 56%, respectively) [18].

In spite of the limited role of PET-CT in establishing the diagnosis of breast cancer, studies have suggested that PET-CT can provide useful histopathological features of cancer, which may have some important influence on planned treatment. Some studies have suggested a positive correlation between FDG uptake, Ki 67 level, and oestrogen receptor (ER) and progesterone receptor (PR) status [19]. In a retrospective study of 548 patients by Koo et al., it was identified that triple-negative and HER2-positive tumours had 1.67-fold (*p* < 0.001) and 1.27-fold (*p* = 0.009) higher SUVmax (standardised uptake value) values, respectively, than luminal A tumours after adjustment for invasive tumour size, lymph node involvement status, and histologic grade on multivariate analysis [20]. Hogan et al., evaluated the usefulness of PET-CT in invasive lobular cancers (ILCs) as these cancers are more difficult to detect than invasive ductal cancers (IDCs) on imaging with a mammogram, ultrasound, and MRI [21,22]. Furthermore, many studies have shown that the ILCs have lower standardised uptake values in comparison with IDCs [23,24]. Hence, metastasis from ILCs may be less appreciable in comparison to metastasis from IDCs [25]. Hogan et al., in a study of 146 ILC patients showed that FDG PET is more likely to identify asymptomatic, clinically occult distant metastasis in stage III IDC than in stage III ILC [21].

### 3.2. PET-CT in Breast Cancer Staging

The current literature has very limited evidence in terms of assessing the clinical usefulness of PET-CT in breast cancer staging and most of the studies evaluating the role of PET are retrospective in nature with limited numbers of patients. 

As many studies have shown that the yield of any staging investigation to diagnose asymptomatic distal metastasis in early-stage breast cancer is very low, the current consensus only advises the use of PET-CT when conventional imaging is equivocal and the patient has stage IIIB breast cancer [11]. However, in a study of 225 patients, Niikura et al., showed that FDG PET-CT has 97.4% sensitivity and 91.2% specificity compared with the 85.9% sensitivity and 67.3% specificity of conventional techniques, including CT, US, and bone scanning, in detecting distant metastases [14]. It is important to note that in this study a good proportion of patients were found to have stage I to stage IIIB breast cancer (41.3%). In another study, Riedl et al., reviewed 134 patients under the age of 40 who underwent FDG PET-CT for staging and found that FDG PET-CT identified unexpected extra-axillary regional nodal and distant metastases in 21% of patients, including 15 patients (11%) showing extra-axillary lymph nodal disease, 20 (15%) showing distant metastases, and 7 cases showing both [15]. It is interesting to see that a significant proportion of these patients had disease stages outside the current guideline recommendations (15% with stage I, 33% with stage IIA, 35% with stage IIB and 17% with stage III) [15].

Bone metastasis is one of the most common types of metastasis in breast cancer. Bone metastasis can be lytic, sclerotic, mixed, or intramedullary, without obvious bone changes [13,26]. FDG-PET is better than bone scanning in identifying lytic and intramedullary metastases, although FDG-PET is less efficient in identifying sclerotic bone metastases. However, these non-avid lesions are often identified in the CT component of FDG-PET scans [27,28].

FDG PET-CT had a sensitivity and negative predictive value of 100% in comparison to a sensitivity of 92% and negative predictive value of 83% obtained with conventional imaging in terms of excluding local recurrence or distant metastases in a study of 77 PET-CT scans in 39 breast cancer patients [28]. The same study showed that PET-CT had a specificity of 76.9% and positive predictive value of 89%, which was comparable to conventional CT, with its specificity of 76.9% and positive predictive value of 88% [28].

### 3.3. PET-CT and Lymph Node Metastases

Currently, the most common image modality used to assess lymph nodes remains US of axilla with a biopsy of abnormal-looking lymph nodes in the preoperative workup of early breast cancer patients. In clinically node-negative patients, a sentinel lymph node biopsy remains the gold standard in terms of staging axilla accurately. Davidson et al., in a study of 324 women with breast cancer demonstrated that FDG PET-CT had a positive predictive value for detecting metastases in axillary lymph nodes of 85.5% and a negative predictive value of 72.9% [29]. Zhang et al., in their meta-analysis of 11 studies showed that FDG PET-CT and MRI had similar sensitivity and specificity in identifying axillary lymph node metastases. The sensitivity of FDG PET-CT ranged from 37 to 90% and specificity ranged from 80 to 100%. Likewise, MRI had a sensitivity range of 40–100% and specificity range of 73–100%. Hence, in the meta-analysis, both FDG PET-CT and MRI had high specificity for axillary lymph node metastases [30]. Zhang et al., suggest that, because of the higher specificity of FDG PET-CT and MRI, these methods can potentially replace sentinel lymph node biopsy in some breast cancer patients. Kutluturk et al., suggested that the accuracy of FDG PET-CT for detecting axillary lymph node metastases is higher with larger tumour sizes [31]. On the contrary, Kim et al., in their series of 262 patients, found that the sensitivity, specificity, and positive and negative predictive values of FDG PET-CT were higher in the subset of patients under the age of 75 years with a tumour size of <15 mm [32]. Parisse-Di Martino et al., reviewed a subset of patients with discordant results with an ultrasound scan and FDG PET-CT to assess axillary lymph nodes from a larger series of 560 breast cancer patients. They found that more than half of the patients in this group with PET-CT displayed axillary lymph node metastases, but with normal ultrasound scanning had an axillary lymph node size of <1 cm [33]. Further, in a recent retrospective study by Kong and Choi (2021), 221 preoperative patients who underwent SLNB and axillary lymph node dissection (ALND) had their FDG PET-CT imaging, histology, and follow-up findings reviewed. The researchers found a positive predictive value of 100% for FDG PET-CT and an ability to detect lymph node involvement with 70% sensitivity [34]. The suggested reasons for false-positive results obtained on PET-CT are previous biopsy, other tumours such as lymphoma, infective and inflammatory conditions, and vaccines [35]. Considering the expense and radiation dose associated with PET-CT, US remains the modality of choice at present for assessing axillary lymph nodes.

Another recent study by Yararbas et al., showed a significant rate of upstaging based on the identification of extra-axillary regional lymph nodes and distant metastases and it was shown that 18.6% of patients with stage IIA, 30% with stage II B, and 46.3% with stage IIIA breast cancer had upstaging after FDG PET-CT [36]. Ko et al., in another study of 195 breast cancer patients with stage II A to stage IIIC disease showed an overall upstaging rate for regional nodal metastases and/or distant metastases of 37% after FDG PET-CT. This included an upstaging of 24% in stage II A, 39% in stage II B, 54% in stage IIIA, 27% in stage IIIB, and 37% in stage IIIC [37]. Seo et al., showed (retrospective study of 249 patients) that FDG PET-CT had a higher positive predictive value (PPV) of 87.1% in diagnosing internal mammary chain lymph node metastasis in stage III cancer [38].

### 3.4. PET-CT and Distant Metastases

The significant benefits of PET-CT are shown in detecting distant metastases. A recent prospective study by Vogsen et al. (2021) found that of the 103 patients enrolled, 23% were diagnosed with distant metastases via [^18^F] FDG-PET-CT. This resulted in surgery being omitted in 18 cases, with 16 patients being upstaged and receiving a subsequent change in treatment. Thus, a sensitivity of 100% and specificity of 95% were demonstrated [39]. This is reflective of the study of Ko et al. (2020), which found that 37% of patients with clinical-stage breast cancer IIA-IIIC who underwent FDG PET-CT had more extensive disease [37]. This included 23% with regional lymph node metastases and 14% with distant metastases, which resulted in a direct upstaging and change of treatment. Bone metastasis is one of the common types of distant metastasis in breast cancer. PET-CT may help to identify focal areas of FDG uptake much earlier than bone scintigraphy. Hansen et al., analysed lesion-based sensitivity of FDG PET-CT, low-dose CT and bone scintigraphy and showed that lesion-based sensitivity was 98.2% and 98.8% for early and delayed FDG PET-CT, respectively, compared with 79.9% for low-dose CT and 76% for bone scanning and 98.6% for combined low-dose CT and bone scanning [40]. In this study, only 51.2% of osteolytic metastases were detected via bone scanning. In another retrospective study of 198 patients, PET-CT showed higher accuracy than CT for detection of bone metastases, demonstrating increased metabolic activity prior to structural changes [41]. Another meta-analysis by Rong et al., consisting of 668 patients in 7 studies shown that PET-CT has a sensitivity of 0.93 and specificity of 0.99 in detecting bone metastases in comparison to a sensitivity of 0.81 and specificity of 0.98 for bone scintigraphy [42].

### 3.5. PET-CT and Prognosis

With the current era of personalised and tailored treatment, prognostic evaluation of breast cancer is important in planning the appropriate management. Many studies have shown that FDG uptake positively correlates with aggressive tumour behaviour and poor prognosis [43,44]. Baba S et al., showed that higher uptake was associated with larger tumours, higher nuclear grade, and triple-negative receptor status [43]. Meta-analysis by Diao et al., of 3574 patients in 15 studies for event-free survival found that patients with higher primary standardised uptake values (SUVmax) showed a poorer survival prognosis, with a pooled HR of 1.96 [45]. Kitajima et al., assessed the relationship between FDG-PET findings and immune microenvironment in breast cancer in a series of 502 patients and found that high SUVmax was related to shorter recurrence-free survival (RFS) than those with low SUVmax in low tumour-infiltration lymphocytes (TIL)patient group. [46].

### 3.6. PET-CT and Treatment Response

The ability to predict responses to neoadjuvant therapy and to identify non-responders early in the treatment would be of great clinical utility in breast cancer management. Currently there is no single gold-standard tool available in our clinical practice. However, a number of studies have shown encouraging results for PET-CT in predicting the response to neoadjuvant systemic therapy. Factors such as higher baseline glycolytic activity and bigger reduction in SUVmax after initial cycles of chemotherapy suggest a pathological response after neoadjuvant chemotherapy [47]. Han S et al., in a recent meta-analysis of 1630 patients in 21 studies showed that a pooled hazard ratio of metabolic responses on disease-free survival was 0.21 for interim PET scans and 0.31 for post-treatment PET scan [48]. The same meta-analysis demonstrated that pooled HRs for interim and post-treatment PET regarding the influence of metabolic responses on overall survival were 0.20 and 0.26, respectively. This suggests that use of PET-CT for the evaluation of response to NAC provides significant predictive value for disease recurrence and survival.

### 3.7. PET-CT and Disease Recurrence

A meta-analysis of 1752 patients in 26 studies with suspicious recurrence of breast cancer by Xiao et al., showed that the pooled sensitivity, specificity, positive likelihood ratio, negative likelihood ratio, and diagnostic odds ratio of FDG PET-CT were 0.90, 0.81, 4.64, 0.12, and 46.52, respectively, and concluded that FDG PET-CT is valuable in detecting cancer relapse. In this meta-analysis, recurrence was suspected because of the elevation of tumour markers (56.8%), suspicion when undergoing conventional imaging modalities (33.9%), and suggestive clinical symptoms or physical examinations (9.4%) [49]. Another study by Hildebrandt et al., showed that, in 100 patients with suspected recurrence, the area under the receiver operating curve for distant recurrence was 0.99 for FDG PET-CT, 0.84 for contrast-enhanced CT, and 0.86 for the combination of contrast-enhanced CT and bone scintigraphy [50]. Vogsen et al., in a prospective study of 225 patients with suspected breast cancer recurrence, showed that the sensitivity, specificity, and AUC-ROC for diagnosing distant metastases via PET-CT were 1.00, 0.88, and 0.98, respectively [51].

Rising tumour markers during post treatment surveillance is a challenging situation to identify breast cancer recurrence. Dong Y et al., in a retrospective study showed that FDG PET-CT was more sensitive in terms of detecting the malignant foci and had better patient-based sensitivity and specificity (95% and 71.4%, respectively) when compared with the sensitivity and specificity of conventional imaging techniques (78.9% and 57.1%) in this setting [52]. Corso et al., retrospectively reviewed 561 breast cancer patients who underwent surgery with curative intent and had raised tumour markers and they found the increased tumour marker levels detected in asymptomatic patients during adjuvant therapies and follow-up to be significantly predictive of distant metastases identified using FDG PET-CT [53].

### 3.8. Impact of Indeterminate Lesions on PET-CT

Even though the accurate staging of breast cancer helps to plan and tailor treatment appropriately, there is variation in diagnostic accuracy for different imaging modalities. FDG PET-CT may have higher accuracy in terms of diagnosing distant metastases than conventional imaging modalities, but it is not completely free from false results. Incidental findings may generate additional tests, causing potential delay in treatment and more importantly anxiety in patients [54]. Vogsen et al., reviewed 225 eligible patients with suspicious cancer recurrence where FDG PET-CT was carried out. In this study, indications for PET-CT were local recurrence in 20% of patients and clinical symptoms in 80% patients. FDG PET-CT was positive for metastases in 32% and negative in 68% patients. A biopsy confirmed metastases in 72.2% of patients with positive FDG PET-CT. Interestingly, 18/225 (8%) patients showed non-breast malignancy on FDG PET-CT [48]. This was similar to the rate of non-breast malignancies identified in other studies [54,55,56,57,58]. FDG-PET-CT provided a high posterior probability of positive test, and a negative test was able to rule out distant metastases in women with clinically suspected recurrent breast cancer. Furthermore, one-fifth of patients examined for incidental findings detected at FDG-PET-CT were diagnosed with clinically relevant conditions. Further examinations of false-positive incidental findings in one of six women should be weighed against the high accuracy for diagnosing metastatic breast cancer [51]. 

### 3.9. PET-CT and Cost Effectiveness

Ko et al., in their study of 195 patients with stage IIA-IIIC breast cancer, compared the cost implications and radiation exposure associated with FDG PET-CT against those with CT of chest, abdomen, and pelvis with bone scan. They found that the costs for both were comparable and with reduced radiation exposure associated with PET-CT [37]. Another recent study by Hyland et al., 564 patients with stage II-III breast cancer data were reviewed to compare the cost implications of staging procedures and concluded that FDG PET-CT reduced false-positive risk by half (22.1% vs. 11.1%) and decreased the workup of incidental findings, allowing for an earlier treatment start, and also found that PET-CT was cost-effective and may be cost-saving in some settings [59].

### 3.10. PET-CT and Future Prospects

The usefulness of PET-CT in breast cancer is mainly limited due to lower sensitivity in terms of identifying smaller tumours of less than 1 cm and low uptake in lower-grade cancers. Studies are evaluating various techniques as well as testing a range of tracers to improve the limitations associated with PET-CT. One of the improved techniques is using total-body PET scanners, which come with ultrahigh sensitivity. This allows them to provide comparable images with significantly lower activity due to a higher signal-to-noise (SNR) ratio. Total-body PET will enable higher sensitivity (up to 68 times higher than PET-CT) and will yield a higher SNR value and allow for a 40-fold reduction in radioactivity dose [60]. It is also reported that total-body PET scans reduce the imaging time by a factor of 24 [60]. Shorter acquisition time also results in less movement-induced blurring. Total-body scanners also address another limitation of PET-CT in identifying smaller lesions, as they are associated with ultrahigh sensitivity, good spatial resolution, and long scan range. Furthermore, the novel, four-dimensional (4D) dynamic whole-body PET acquisition method improves tumour characterisation [61]. Another advantage of a total-body scanner is the 10-fold reduction in the file size of raw PET data, permitting faster data processing, reconstruction, and transport [62]. A longer acquisition delay permits researchers to carry out scans at later time points after tracer injection and this may be helpful in identifying smaller lesions and cancers with low avidity. Another major advantage of total-body scanners is their ability to differentiate between residual disease and post-therapy changes. Another important development to address limitations of PET based imaging is to implement multi-tracer PET studies using cocktail injections where two radiopharmaceuticals are injected prior to a single PET acquisition [63]. Sodium fluoride (NaF) reflects osteoblastic activity with high potential for detecting osteoblastic metastases when combined with FDG. New PET tracers such as ^89^Zr-trastuzumab and ^89^Zr-pertuzumab were developed for measuring Her2 expression in the primary and metastatic lesions non-invasively [64]. 

#### 3.10.1. Artificial Intelligence and PET-CT Radiomics

**Figure 1:**
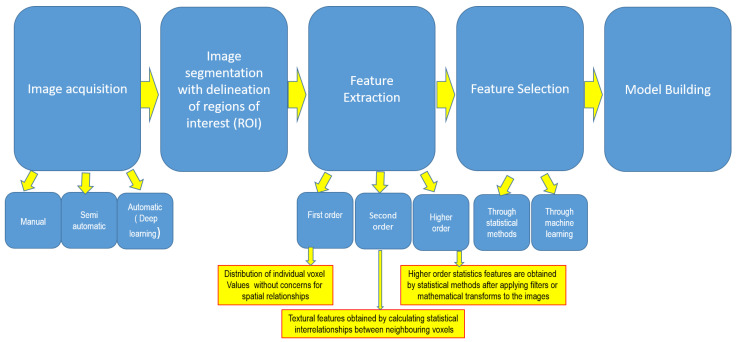
Steps of radiomics and artificial intelligence workflow.

With the paradigm shift towards personalised medicine, the identification of reliable and non-invasive biomarkers able to predict tumour heterogeneity is pivotal in improving patient treatment. At present, tumour biology is deciphered using invasive procedures such as biopsy, which has limitations. Biopsy results from one lesion or one part of the lesion may not necessarily represent the whole-tumour heterogeneity [65,66]. Another limitation of using invasive biopsy to identify tumour biology is the inability to sample all suspicious distant metastatic lesions in order to identify any clonal difference. Radiomics is an emerging technique in the field of medical image analysis and is used to assess tumour biology non-invasively by identifying mineable variables hidden in the pixels of images not routinely visualised by the human eye (Figure 1). This helps to avoid the requirement multiple and repeated biopsies to aid treatment planning in breast cancer [67,68,69]. 

Current research into artificial intelligence (AI) and radiomics in the area of PET-CT and other areas of medical imaging focuses on the interpretation of images for diagnosis and staging, a task which, when traditionally conducted by humans, it is known to be time-consuming and subject to observer variability [70].

With the current development of artificial intelligence, the development of algorithms, tools and applications is rapidly evolving in the field of nuclear medicine [71]. A study by Yoon et al., carried out a texture-based analysis of intratumoural metabolic heterogeneity to identify the presence of invasive components in a retrospective analysis of 65 patients with ductal carcinoma in situ (DCIS) who underwent FDG PET-CT. They found that a lower area under the curve (AUC) of cumulative SUV histograms, a parameter reflecting higher intratumoural heterogeneity, was associated with the underestimation of invasive disease and suggested sentinel lymph node biopsy in this subset of patients [72]. A number of studies have already analysed a range of radiomic parameters to predict tumour biology with variable results [73,74,75,76,77,78]. Several studies have evaluated the potential use of PET-generated radiomic features and artificial intelligence to predict the response to neoadjuvant chemotherapy with varying degrees of success [74,76,79]. Song and colleagues proposed a machine learning (ML)-based radiomic model, developed by analysing FDG PET-CT, with a view to predicting axillary lymph node metastases in a study of 100 patients with invasive ductal breast cancer and demonstrated that the model showed 90.9% sensitivity, 71.4% specificity, and 80% accuracy in the preoperative detection of axillary lymph node metastases [80]. Another potential area where AI and PET radiomics will be useful is in assessing treatment response, especially in patients with multiple metastases. The manual segmentation of all metastatic lesions is time-consuming. Moreau and colleagues showed very promising results in this field by training two deep learning models to automatically segment metastatic lesions on the baseline and follow up PET-CT in 60 patients with 87% sensitivity and 87% specificity in terms of assessing treatment response [81]. Huang et al., and Ha et al., applied AI to FDG PET to obtain prognostic data and showed the good correlation of radiomic variables and tumour molecular subtypes, immunohistochemistry, and relapse-free survival [74,82]. 

Dedicated breast PET (dbPET) provides high-resolution molecular imaging acquired from uncompressed breast tissue using a high-resolution full-ring dedicated breast tomograph and a study by Satoh et al., showed that a deep learning model had been trained to 93% sensitivity and specificity in comprehending breast cancer and non-breast cancer in 160 breasts, compared with 77–89% sensitivity and 79–100% specificity obtained from two expert radiologists [83]. PET radiomics has the potential to improve diagnosis, staging, pathological characterisation, treatment response assessment, and prognostication in breast cancer patients [84].

#### 3.10.2. Novel Tracers in PET-CT

Breast cancer is a very heterogeneous disease and a number of biomarkers such as oestrogen receptor (ER), progesterone receptor (PR), Human Epidermal Growth Factor Receptor 2 (Her2) and Ki-67 do have influence on ideal treatment options and prognosis. There are a several limitations with the current means of assessment of histological characteristics and biomarkers based on invasive biopsy techniques. It is not always possible or practical to identify the receptor status of the disease, especially when the lesions are difficult to biopsy in the metastatic setting. It is also not uncommon to see that the receptor status of secondary lesions may be different from that of the primary tumour and it is not feasible to biopsy every new lesion that appears. A number of studies have tried using receptor-specific nuclear imaging techniques to assess tumour characteristics non-invasively. 

2-[^18^F]-fluoro-2-deoxy-D-glucose ([^18^F]F-FDG) represents the most widely used radiopharmaceutical for PET imaging to date. Glucose metabolism is increased in breast cancer cells compared with normal cells due to increased glycolysis (Warburg effect). However, this technique has a number of limitations such as difficulty in appropriate interpretation in small tumours because of the low spatial resolution of PET tomographs, the partial volume effect, and the low sensitivity for certain tumour types due to low avidity [85]. Furthermore, [^18^F]F-FDG is not a specific radiotracer for cancer cells and other conditions such as infection, inflammation, benign lumps, and fibrocystic changes can also lead to false-positive results. 

In breast cancer, increased protein synthesis is associated with increased amino acid consumption and the overexpression of amino acid transporters in the cell membrane. L-methyl-[^11^C]-methionine ([^11^C]C-MET) represents one of the first radiolabeled amino acids used for the assessment of amino acid metabolism in oncologic PET imaging. Studies have shown that [^11^C]C-MET uptake was reduced in responsive lesions, while it was unchanged or even increased in patients with progressing disease and hence may have a role in assessing treatment responses in breast cancer patients [86]. Other [^18^F]-labeled amino acids such as Anti-1-amino-3-[18F]-fluorocyclo-butane-1-carboxylic acid ([^18^F]-fluciclovine or [^18^F]F-FACBC) is also useful in breast cancer patients. Interestingly, two different studies showed that [^18^F]F-FACBC avidity is higher than [^18^F]FFDG avidity in invasive lobular cancer and equal to [^18^F]F-FDG avidity in invasive ductal cancer [87,88]. 

The presence of hypoxia in many tumor types, including breast cancer, has been shown to induce resistance to both chemotherapy and radiation therapy, representing a negative prognostic factor. Radiolabeled nitroimidazoles represent the most widely developed hypoxia probe for PET imaging in oncology [86]. 

[^18^F]-labeled estradiol is used for in vivo assessment of oestrogen receptor status in both primary and metastatic lesions. Meta-analysis by Kurland et al., demonstrated that [^18^F]F-FES non-invasively characterizes the ER ligand binding function in breast cancer lesions with a sensitivity of 0.81 (0.73–0.87) and a specificity of 0.86 (0.68–0.94) compared to the histological standard of reference [89]. The main disadvantages of [^18^F]F-FES include high uptake in the liver, making it difficult to assess liver metastasis, and rapid blood clearance, which can lead to lower tumoral uptake.

[^89^Zr]Zr-trastuzumab is a PET imaging radiopharmaceutical technique capable of qualitatively and quantitatively assessing HER2 expression in both primary and metastatic lesions in breast cancer patients. Long half-life of [^89^Zr] can lead to increased radiation exposure. Recent studies have shown that [^89^Zr]Zr-trastuzumab PET-CT supports clinical decision making when HER2 status cannot be determined by biopsy [90]. [^64^Cu]Cu-DOTA-trastuzumab is another tracer for the Her2 marker but with shorter half-life and reduced radiation exposure.

With the increasing use of immunotherapy with immune-check point inhibitors in certain subsets of breast cancers such as triple-negative breast cancer, it is important to identify new biomarkers that can predict response and resistance to immunotherapy. New PET tracers targeting immune checkpoint proteins such as [^89^Zr]Zr-atezolizumab are currently under evaluation in this setting [91].

## 4. Conclusions

There is a growing body of evidence to support the clinical usefulness of PET-CT in early and locally advanced breast cancer. Accurate staging information and tumour characterisation are important in tailoring appropriate treatment for breast cancer patients. The limited studies assessing the cost evaluation suggest that PET-CT is cost effective as a staging modality. Furthermore, ongoing research in PET imaging techniques and tracers to address the current limitations of PET CT is encouraging. Ongoing research in the field of PET derived radiomics, artificial intelligence, and new tracers is very promising, especially in tumour characterisation, evaluating lymph node status, and predicting responses to neoadjuvant chemotherapy. At present, PET radiomic studies are still non-standardised, lack reproducibility, and need further validation. Larger prospective studies are needed to confirm the clinical utility and effectiveness of PET imaging in diagnosis, staging, pathological characterisation, prognostication, as well as treatment response assessment in future.

## References

[1] Siegel R.L., Miller K.D., Jemal A. (2018). Cancer statistics, 2018. CA A Cancer J. Clin..

[2] Groheux D. (2022). FDG-PET/CT for Primary Staging and Detection of Recurrence of Breast Cancer. Semin. Nucl. Med..

[3] Cancer Research UK (2023). Breast Cancer Survival Statistics. https://web.archive.org/web/20220207071809/https://www.cancerresearchuk.org/health-professional/cancer-statistics/statistics-by-cancer-type/breast-cancer/survival.

[4] James J., Teo M., Ramachandran V., Law M., Stoney D., Cheng M. (2017). Performance of CT scan of abdomen and pelvis in detecting asymptomatic synchronous metastasis in breast cancer. Int. J. Surg..

[5] National Cancer Registry Breast Cancer Incidence, Mortality, Treatment and Survival in Ireland: 1994–2009. [Internet]. http://www.ncri.ie/publications/statistical-reports/breast-cancer.

[6] Lyratzopoulos G., Abel G.A., Barbiere J.M., Brown C.H., Rous B.A., Greenberg D.C. (2012). Variation in advanced stage at diagnosis of lung and female breast cancer in an English region 2006–2009. Br. J. Cancer.

[7] Johnson R.H., Chien F.L., Bleyer A. (2013). Incidence of Breast Cancer with Distant Involvement among Women in the United States, 1976 to 2009. JAMA.

[8] Costelloe C.M., Rohren E.M., Madewell J.E., Hamaoka T., Theriault R.L., Yu T.-K., Lewis V.O., Ma J., Stafford R.J., Tari A.M. (2009). Imaging bone metastases in breast cancer: Techniques and recommendations for diagnosis. Lancet Oncol..

[9] Brennan M.E., Houssami N. (2012). Evaluation of the evidence on staging imaging for detection of asymptomatic distant metastases in newly diagnosed breast cancer. Breast.

[10] National Institute for Health and Care Excellence (2018). Early and Locally Advanced Breast Cancer: Diagnosis and Management. https://www.nice.org.uk/guidance/ng101.

[11] National Comprehensive Cancer Network (2023). NCCN Guidelines Breast Cancer. https://www.nccn.org/guidelines/recently-published-guidelines.

[12] Loibl S., André F., Bachelot T., Barrios C.H., Bergh J., Burstein H.J., Cardoso M.J., Carey L.A., Dawood S., Del Mastro L. (2024). Early Breast Cancer: ESMO Clinical Practice Guideline for Diagnosis, Treatment and Follow-Up. Ann. Oncol..

[13] Abouzied M.M., Fathala A., AlMuhaideb A., Al Qahtani M.H. (2020). Role of ^18^F-fluorodeoxyglucose positron emission tomography/computed tomography in the evaluation of breast carcinoma: Indications and pitfalls with illustrative case examples. World J. Nucl. Med..

[14] Niikura N., Costelloe C.M., Madewell J.E., Hayashi N., Yu T.-K., Liu J., Palla S.L., Tokuda Y., Theriault R.L., Hortobagyi G.N. (2011). FDG-PET/CT compared with conventional imaging in the detection of distant metastases of primary breast cancer. Oncologist.

[15] Riedl C.C., Slobod E., Jochelson M., Morrow M., Goldman D.A., Gonen M., Weber W.A., Ulaner G.A. (2014). Retrospective analysis of 18F-FDG PET/CT for staging asymptomatic breast cancer patients younger than 40 years. J. Nucl. Med..

[16] Kumar R., Chauhan A., Zhuang H., Chandra P., Schnall M., Alavi A. (2006). Clinicopathologic factors associated with false negative FDG-PET in primary breast cancer. Breast Cancer Res. Treat..

[17] Warning K., Hildebrandt M.G., Kristensen B., Ewertz M. (2018). Utility of 18FDG-PET/CT in breast cancer diagnostics—A systematic review. Dan. Med. Bull..

[18] Grueneisen J., Nagarajah J., Buchbender C., Hoffmann O., Schaarschmidt B.M., Poeppel T., Forsting M., Quick H.H., Umutlu L., Kinner S. (2015). Positron emission tomography/magnetic resonance imaging for local tumor staging in patients with primary breast cancer: A comparison with positron emission tomography/computed tomography and magnetic resonance imaging. Investig. Radiol..

[19] Miyake K.K., Nakamoto Y., Kanao S., Tanaka S., Sugie T., Mikami Y., Toi M., Togashi K. (2014). Journal Club: Diagnostic value of 18F-FDG PET/CT and MRI in predicting the clinicopathologic subtypes of invasive breast cancer. Am. J. Roentgenol..

[20] Koo H.R., Park J.S., Kang K.W., Cho N., Chang J.M., Bae M.S., Kim W.H., Lee S.H., Kim M.Y., Kim J.Y. (2014). ^18^F-FDG uptake in breast cancer correlates with immunohistochemically defined subtypes. Eur. Radiol..

[21] Hogan M.P., Goldman D.A., Dashevsky B., Riedl C.C., Gönen M., Osborne J.R., Jochelson M., Hudis C., Morrow M., Ulaner G.A. (2015). Comparison of ^18^F-FDG PET/CT for Systemic Staging of Newly Diagnosed Invasive Lobular Carcinoma Versus Invasive Ductal Carcinoma. J. Nucl. Med..

[22] Lopez J.K., Bassett L.W. (2009). Invasive lobular carcinoma of the breast: Spectrum of mammographic US, and MR imaging findings. Radiographics.

[23] Avril N., Menzel M., Dose J., Schelling M., Weber W., Jänicke F., Nathrath W., Schwaiger M. (2001). Glucose metabolism of breast cancer assessed by 18F-FDG PET: Histologic and immunohistochemical tissue analysis. J. Nucl. Med..

[24] Bos R., Van der Hoeven J.J., Van der Wall E., Van der Groep P., Van Diest P.J., Comans E.F., Joshi U., Semenza G.L., Hoekstra O.S., Lammertsma A.A. (2002). Biologic correlates of 18fluorodeoxyglucose uptake in human breast cancer measured by positron emission tomography. J. Clin. Oncol..

[25] Dashevsky B.Z., Goldman D.A., Parsons M., Gönen M., Corben A.D., Jochelson M.S., Hudis C.A., Morrow M., Ulaner G.A. (2015). Appearance of untreated bone metastases from breast cancer on FDG PET/CT: Importance of histologic subtype. Eur. J. Nucl. Med. Mol. Imaging.

[26] Cook G.J., Houston S., Rubens R., Maisey M.N., Fogelman I. (1998). Detection of bone metastases in breast cancer by ^18^FDG PET: Differing metabolic activity in osteoblastic and osteolytic lesions. J. Clin. Oncol..

[27] Nakai T., Okuyama C., Kubota T., Yamada K., Ushijima Y., Taniike K., Suzuki T., Nishimura T. (2005). Pitfalls of FDG-PET for the diagnosis of osteoblastic bone metastases in patients with breast cancer. Eur. J. Nucl. Med. Mol. Imaging.

[28] Algafri A., Al-Tweigeri T., Al-Sugair A., Al-Seabee M., Al-Alawi E., Fathala A., Almuhaideb A., Tuli M., Abouzied M. (2012). The Diagnostic Accuracy of FEG PET Low Dose Non Enhanced CT in Detection of Local Recurrence and Distant Metastases during Follow-up of Breast Cancer Patients: A Comparison to Enhanced CT and Bone Scan. Proceedings of the European Congress of Radiology 2012.

[29] Davidson T., Shehade Nissan E., Sklair-Levy M., Ben-Haim S., Barshack I., Zippel D., Halevy A., Chikman B. (2021). PET/CT in breast cancer staging is useful for evaluation of axillary lymph node and distant metastases. Surg. Oncol..

[30] Zhang X., Liu Y., Luo H., Zhang J. (2020). PET/CT and MRI for Identifying Axillary Lymph Node Metastases in Breast Cancer Patients: Systematic Review and Meta-Analysis. J. Magn. Reson. Imaging.

[31] Kutluturk K., Simsek A., Comak A., Gonultas F., Unal B., Kekilli E. (2019). Factors affecting the accuracy of ^18^F-FDG PET/CT in evaluating axillary metastases in invasive breast cancer. Niger. J. Clin. Pract..

[32] Kim J., Cho H., Gwak G., Yang K., Kim J.-Y., Shin Y.-J., Seo Y.Y., Park I. (2020). Factors affecting the negative predictive value of positron emission tomography/computed tomography for axillary lymph node staging in breast cancer patients. Asian J. Surg..

[33] Parisse-Di Martino S., Faure C., Mognetti T. (2021). Discordant results in ^18^F-FDG PET/CT and ultrasound-based assessment for axillary lymph node metastasis detection: A large retrospective analysis in 560 patients with breast cancer. Cancer Treat. Res. Commun..

[34] Kong E., Choi J. (2021). The new perspective of PET/CT for axillary nodal staging in early breast cancer patients according to ACOSOG Z0011 trial PET/CT axillary staging according to Z0011. Nucl. Med. Commun..

[35] Brown A.H., Shah S., Groves A.M., Wan S., Malhotra A. (2021). The Challenge of Staging Breast Cancer with PET/CT in the Era of COVID Vaccination. Clin. Nucl. Med..

[36] Yararbas U., Çetin N., Yeniay L., Argon A.M. (2018). The value of ^18^F-FDG PET/CT imaging in breast cancer staging. Bosn. J. Basic Med Sci..

[37] Ko H., Baghdadi Y., Love C., Sparano J.A. (2020). Clinical Utility of ^18^F-FDG PET/CT in Staging Localized Breast Cancer Before Initiating Preoperative Systemic Therapy. J. Natl. Compr. Cancer Netw..

[38] Seo M.J., Lee J.J., Kim H.O., Chae S.Y., Park S.H., Ryu J.S., Ahn S.H., Lee J.W., Son B.H., Gong G.Y. (2014). Detection of internal mammary lymph node metastasis with ^18^F-fluorodeoxyglucose positron emission tomography/computed tomography in patients with stage III breast cancer. Eur. J. Nucl. Med. Mol. Imaging.

[39] Vogsen M., Jensen J.D., Christensen I.Y., Gerke O., Jylling A.M.B., Larsen L.B., Braad P.E., Søe K.L., Bille C., Ewertz M. (2021). FDG-PET/CT in high-risk primary breast cancer-a prospective study of stage migration and clinical impact. Breast Cancer Res. Treat..

[40] Hansen J.A., Naghavi-Bezhad M., Gerke O., Baun C., Falch K., Duvnjak S., Alavi A., Hoilund-Carlsen P.F., Hildebrandt M.G. (2021). Diagnosis of bone metastases in breast cancer: Lesion-based sensitivity of dual-time-point FDG-PET/CT compared to low-dose CT and bone scintigraphy. PLoS ONE.

[41] Evangelista L., Panunzio A., Polverosi R., Ferretti A., Chondrogiannis S., Pomerri F., Rubello D., Muzzio P.C. (2012). Early bone marrow metastasis detection: The additional value of FDG-PET/CT vs. CT imaging. Biomed. Pharmacother..

[42] Rong J., Wang S., Ding Q., Yun M., Zheng Z., Ye S. (2013). Comparison of 18 FDG PET-CT and bone scintigraphy for detection of bone metastases in breast cancer patients. A meta-analysis. Surg. Oncol..

[43] Baba S., Isoda T., Maruoka Y., Kitamura Y., Sasaki M., Yoshida T., Honda H. (2014). Diagnostic and prognostic value of pretreatment SUV in ^18^F-FDG/PET in breast cancer: Comparison with apparent diffusion coefficient from diffusion weighted MR imaging. J. Nucl. Med..

[44] Chang C.C., Tu H.P., Chen Y.W., Lin C.Y., Hou M.F. (2014). Tumour and lymph node uptakes on dual-phased 2-deoxy-2-[^18^F]fluoro-D-glucose positron emission tomography/computed tomography correlate with prognostic parameters in breast cancer. J. Int. Med. Res..

[45] Diao W., Tian F., Jia Z. (2018). The prognostic value of SUVmax measuring on primary lesion and ALN by ^18^F-FDG PET or PET/CT in patients with breast cancer. Eur. J. Radiol..

[46] Kitajima K., Higuchi T., Fujimoto Y., Ishikawa E., Yokoyama H., Komoto H., Inao Y., Yamakado K., Miyoshi Y. (2023). Relationship between FDG-PET and the immune microenvironment in breast cancer. Eur. J. Radiol..

[47] Groheux D., Giacchetti S., Delord M., de Roquancourt A., Merlet P., Hamy A.S., Espié M., Hindié E. (2015). Prognostic impact of ^18^F-FDG PET/CT staging and of pathological response to neoadjuvant chemotherapy in triple-negative breast cancer. Eur. J. Nucl. Med. Mol. Imaging.

[48] Han S., Choi J.Y. (2020). Prognostic value of ^18^F-FDG PET and PET/CT for assessment of treatment response to neoadjuvant chemotherapy in breast cancer: A systematic review and metaanalysis. Breast Cancer Res..

[49] Xiao Y., Wang L., Jiang X., She W., He L., Hu G. (2016). Diagnostic efficacy of ^18^F-FDG-PET or PET/CT in breast cancer with suspected recurrence: A systematic review and meta-analysis. Nucl. Med. Commun..

[50] Hildebrandt M.G., Gerke O., Baun C., Falch K., Hansen J.A., Farahani Z.A., Petersen H., Larsen L.B., Duvnjak S., Buskevica I. (2016). [^18^F]Fluorodeoxyglucose (FDG)-Positron Emission Tomography (PET)/Computed Tomography (CT) in suspected recurrent breast cancer: A prospective comparative study of dual-time-point FDG-PET/CT, Contrast-Enhanced CT, and bone scintigraphy. J. Clin. Oncol..

[51] Vogsen M., Jensen J.D., Gerke O., Jylling A.M.B., Asmussen J.T., Christensen I.Y., Braad P.E., Thye-Rønn P., Søe K.L., Ewertz M. (2021). Benefits and harms of implementing [^18^F]FDG-PET/CT for diagnosing recurrent breast cancer: A prospective clinical study. EJNMMI Res..

[52] Dong Y., Hou H., Wang C., Li J., Yao Q., Amer S., Tian M. (2015). The Diagnostic Value of ^18^F-FDG PET/CT in Association with Serum Tumor Marker Assays in Breast Cancer Recurrence and Metastasis. BioMed Res. Int..

[53] Corso G., Gilardi L., Girardi A., De Scalzi A.M., Pagani G., Rossi E.M.C., Montagna G., Veronesi P., Pagan E., Bagnardi V. (2020). How useful are tumor markers in detecting metastases with FDG-PET/CT during breast cancer surveillance?. Oncology.

[54] Ishimori T., Patel P.V., Wahl R.L. (2005). Detection of unexpected additional primary malignancies with PET/CT. J. Nucl. Med. Off. Publ. Soc. Nucl. Med..

[55] Britt C.J., Maas A.M., Kennedy T.A., Hartig G.K. (2018). Incidental findings on FDG PET/CT in head and neck cancer. Otolaryngol. Head. Neck Surg..

[56] Kousgaard S.J., Thorlacius-Ussing O. (2017). Incidental colorectal FDG uptake on PET/CT scan and lesions observed during subsequent colonoscopy: A systematic review. Tech. Coloproctol..

[57] Rohde M., Nielsen A.L., Johansen J., Sørensen J.A., Nguyen N., Diaz A., Nielsen M.K., Asmussen J.T., Christiansen J.M., Gerke O. (2017). Head-to-head comparison of chest X-ray/head and neck MRI, chest CT/head and neck MRI, and (^18^)F-FDG PET/CT for detection of distant metastases and synchronous cancer in oral, pharyngeal, and laryngeal cancer. J. Nucl. Med. Off. Publ. Soc. Nucl. Med..

[58] Sponholtz S.E., Mogensen O., Hildebrandt M.G., Jensen P.T. (2020). Clinical impact of pre-treatment FDG-PET/CT staging of primary ovarian, fallopian tube, and peritoneal cancers in women. Acta Obstet. Gynecol. Scand..

[59] Hyland C.J., Varghese F., Yau C., Beckwith H., Khoury K., Varnado W., Hirst G.L., Flavell R.R., Chien A.J., Yee D. (2020). Use of ^18^F-FDG PET/CT as an Initial Staging Procedure for Stage II–III Breast Cancer: A Multicenter Value Analysis. J. Natl. Compr. Cancer Netw..

[60] Katal S., Eibschutz L.S., Saboury B., Gholamrezanezhad A., Alavi A. (2022). Advantages and Applications of Total-Body PET Scanning. Diagnostics.

[61] Sui X., Liu G., Hu P., Chen S., Yu H., Wang Y., Shi H. (2021). Total-Body PET/Computed Tomography Highlights in Clinical Practice. PET Clin..

[62] Lan X., Fan K., Li K., Cai W. (2021). Dynamic PET imaging with ultra-low-activity of 18F-FDG: Unleashing the potential of total-body PET. Eur. J. Nucl. Med. Mol. Imaging.

[63] Roop M.J., Singh B., Singh H., Watts A., Kohli P.S., Mittal B.R., Singh G. (2017). Incremental Value of Cocktail ^18^F-FDG and ^18^F-NaF PET/CT over ^18^F-FDG PET/CT Alone for Characterization of Skeletal Metastases in Breast Cancer. Clin. Nucl. Med..

[64] Ulaner G.A., Hyman D.M., Lyashchenko S.K., Lewis J.S., Carrasquillo J.A. (2017). 89Zr-Trastuzumab PET/CT for detection of human epidermal growth factor receptor 2-positive metastases in patients with human epidermal growth factor receptor 2-negative primary breast cancer. Clin. Nucl. Med..

[65] Haynes B., Sarma A., Nangia-Makker P., Shekhar M.P. (2017). Breast cancer complexity: Implications of intratumoral heterogeneity in clinical management. Cancer Metastasis Rev..

[66] Cajal S.R.Y., Sesé M., Capdevila C., Aasen T., Mattos-Arruda L., Diaz-Cano S.J., Hernández-Losa J., Castellví J. (2020). Clinical implications of intratumor heterogeneity: Challenges and opportunities. J. Mol. Med..

[67] Castello A., Castellani M., Florimonte L., Urso L., Mansi L., Lopci E. (2022). The Role of Radiomics in the Era of Immune Checkpoint Inhibitors: A New Protagonist in the Jungle of Response Criteria. J. Clin. Med..

[68] Ibrahim A., Primakov S., Beuque M., Woodruff H., Halilaj I., Wu G., Refaee T., Granzier R., Widaatalla Y., Hustinx R. (2021). Radiomics for precision medicine: Current challenges, future prospects, and the proposal of a new framework. Methods.

[69] Lambin P., Leijenaar R.T.H., Deist T.M., Peerlings J., de Jong E.E.C., van Timmeren J., Sanduleanu S., Larue R.T.H.M., Even A.J.G., Jochems A. (2017). Radiomics: The bridge between medical imaging and personalized medicine. Nat. Rev. Clin. Oncol..

[70] Fallahpoor M., Chakraborty S., Pradhan B., Faust O., Barua P.D., Chegeni H., Acharya R. (2024). Deep learning techniques in PET/CT imaging: A comprehensive review from sinogram to image space. Comput. Methods Programs Biomed..

[71] Aktolun C. (2019). Artificial intelligence and radiomics in nuclear medicine: Potentials and challenges. Eur. J. Nucl. Med. Mol. Imaging.

[72] Yoon H.J., Kim Y., Kim B.S. (2015). Intratumoral metabolic heterogeneity predicts invasive components in breast ductal carcinoma in situ. Eur. Radiol..

[73] Lemarignier C., Martineau A., Teixeira L., Vercellino L., Espié M., Merlet P., Groheux D. (2017). Correlation between tumour characteristics, SUV measurements, metabolic tumour volume, TLG and textural features assessed with ^18^F-FDG PET in a large cohort of oestrogen receptor-positive breast cancer patients. Eur. J. Nucl. Med. Mol. Imaging.

[74] Ha S., Park S., Bang J.I., Kim E.K., Lee H.Y. (2017). Metabolic Radiomics for Pretreatment ^18^F-FDG PET/CT to Characterize Locally Advanced Breast Cancer: Histopathologic Characteristics, Response to Neoadjuvant Chemotherapy, and Prognosis. Sci. Rep..

[75] Aide N., Elie N., Blanc-Fournier C., Levy C., Salomon T., Lasnon C. (2021). Hormonal Receptor Immunochemistry Heterogeneity and ^18^F-FDG Metabolic Heterogeneity: Preliminary Results of Their Relationship and Prognostic Value in Luminal Non-Metastatic Breast Cancers. Front. Oncol..

[76] Molina-García D., García-Vicente A.M., Pérez-Beteta J., Amo-Salas M., Martínez-González A., Tello-Galán M.J., Soriano-Castrejón Á., Pérez-García V.M. (2018). Intratumoral heterogeneity in (^18^)F-FDG PET/CT by textural analysis in breast cancer as a predictive and prognostic subrogate. Ann. Nucl. Med..

[77] Yoon H.J., Kim Y., Chung J., Kim B.S. (2019). Predicting neo-adjuvant chemotherapy response and progression-free survival of locally advanced breast cancer using textural features of intratumoral heterogeneity on F-18 FDG PET/CT and diffusion-weighted MR imaging. Breast J..

[78] Park J.E., Kim D., Kim H.S., Park S.Y., Kim J.Y., Cho S.J., Shin J.H., Kim J.H. (2020). Quality of science and reporting of radiomics in oncologic studies: Room for improvement according to radiomics quality score and TRIPOD statement. Eur. Radiol..

[79] Li P., Wang X., Xu C., Liu C., Zheng C., Fulham M.J., Feng D., Wang L., Song S., Huang G. (2020). ^18^F-FDG PET/CT radiomic predictors of pathologic complete response (pCR) to neoadjuvant chemotherapy in breast cancer patients. Eur. J. Nucl. Med. Mol. Imaging.

[80] Song B.-I. (2021). A machine learning-based radiomics model for the prediction of axillary lymph-node metastasis in breast cancer. Breast Cancer.

[81] Moreau N., Rousseau C., Fourcade C., Santini G., Brennan A., Ferrer L., Lacombe M., Guillerminet C., Colombié M., Jézéquel P. (2022). Automatic segmentation of metastatic breast cancer lesions on18f-fdg pet/ct longitudinal acquisitions for treatment response assessment. Cancers.

[82] Huang S., Franc B.L., Harnish R.J., Liu G., Mitra D., Copeland T.P., Arasu V.A., Kornak J., Jones E.F., Behr S.C. (2018). Exploration of PET and MRI radiomic features for decoding breast cancer phenotypes and prognosis. Npj Breast Cancer.

[83] Satoh Y., Imokawa T., Fujioka T., Mori M., Yamaga E., Takahashi K., Takahashi K., Kawase T., Kubota K., Tateishi U. (2022). Deep learning for image classification in dedicated breast positron emission tomography (dbPET). Ann. Nucl. Med..

[84] Urso L., Manco L., Castello A., Evangelista L., Guidi G., Massimo Castellani M., Florimonte L., Cittanti C., Turra A., Panareo S. (2022). PET-Derived Radiomics and Artificial Intelligence in Breast Cancer: A Systematic Review. Int. J. Mol. Sci..

[85] Balma M., Liberini V., Racca M., Laudicella R., Bauckneht M., Buschiazzo A., Nicolotti D.G., Peano S., Bianchi A., Albano G. (2022). Non-conventional and Investigational PET Radiotracers for Breast Cancer: A Systematic Review. Front. Med..

[86] Ulaner G.A., Schuster D.M. (2018). Amino acid metabolism as a target for breast cancer imaging. PET Clin..

[87] Ulaner G.A., Goldman D.A., Gönen M., Pham H., Castillo R., Lyashchenko S.K., Lewis J.S., Dang C. (2016). Initial results of a prospective clinical trial of 18FFluciclovine PET/CT in newly diagnosed invasive ductal and invasive lobular breast cancers. J. Nucl. Med..

[88] Tade F.I., Cohen M.A., Styblo T.M., Odewole O.A., Holbrook A.I., Newell M.S., Savir-Baruch B., Li X., Goodman M.M., Nye J.A. (2016). Anti-3-18F-FACBC (18F-Fluciclovine) PET/CT of breast cancer: An exploratory study. J. Nucl. Med..

[89] Kurland B.F., Wiggins J.R., Coche A., Fontan C., Bouvet Y., Webner P., Divgi C., Linden H.M. (2020). Whole-body characterization of estrogen receptor status in metastatic breast cancer with 16a-18F-Fluoro-17b-estradiol positron emission tomography: Meta-analysis and recommendations for integration into clinical applications. Oncologist.

[90] Bensch F., Brouwers A.H., Hooge M.N.L.-D., de Jong J.R., van der Vegt B., Sleijfer S., de Vries E.G.E., Schröder C.P. (2018). 89Zr-trastuzumab PET supports clinical decision making in breast cancer patients, when HER2 status cannot be determined by standard work up. Eur. J. Nucl. Med. Mol. Imaging.

[91] Bensch F., Van der veen E.L., Lub-de Hooge M.N., Jorritsma-Smit A., Boellaard R., Kok I.C., Oosting S.F., Schröder C.P., Hiltermann T.J.N., Van Der Wekken A.J. (2018). 89Zr-atezolizumab imaging as a non-invasive approach to assess clinical response to PD-L1 blockade in cancer. Nat. Med..

